# The relevance of body mass index in forensic age assessment of living individuals: an age-adjusted linear regression analysis using multivariable fractional polynomials

**DOI:** 10.1007/s00414-020-02381-2

**Published:** 2020-07-23

**Authors:** Maximilian Timme, André Karch, Denys Shay, Christian Ottow, Andreas Schmeling

**Affiliations:** 1 Institute of Legal Medicine, University Hospital Muenster, Röntgenstraße 23, 48149 Münster, Germany; 2 Institute of Epidemiology and Social Medicine, University of Muenster, Domagkstraße 3, 48149 Münster, Germany; 3 Department of Clinical Radiology, University Hospital Muenster, Albert-Schweitzer-Campus 1, 48149 Münster, Germany

**Keywords:** Age assessment, Body mass index, Minimum-age concept, Bone age

## Abstract

In forensic age assessment of living individuals, developmental stages of skeletal maturation and tooth mineralization are examined and compared with a reference population. It is of interest which factors can affect the development of these features. We investigated the effect of body mass index (BMI) on the developmental stages of the medial epiphysis of the clavicle, the distal epiphysis of the radius, the distal epiphysis of the femur, the proximal epiphysis of the tibia, and the left lower third molar in a total of 581 volunteers, 294 females and 287 males aged 12–24 years, using 3 T MRI. BMI values in the cohort ranged from 13.71 kg/m^2^ in a 12-year-old female to 35.15 kg/m^2^ in an 18-year-old female. The effect of BMI on the development of the characteristics was investigated using linear regression models with multivariable fractional polynomials. In the univariable analysis, BMI was associated with all feature systems (beta between 0.10 and 0.44; *p* < 0.001). When accounting for the physiological increase of BMI with increasing age, the effect of BMI was lower and in the majority of the models no longer clinically relevant. Betas decreased to values between 0.00 and 0.05. When adding feature variables to a model already including age, *r*^2^ values increased only minimally. For an overall bone ossification score combining all characteristics, the adjusted *ß* was 0.11 (*p* = 0.021) and 0.08 (*p* = 0.23) for females and males, respectively. Low *ß* and *r*^2^ values (0.00 (adjusted)–0.16 (crude)) were present in both models for third molar development already in the unadjusted analyses. In conclusion, our study found no to little effect of BMI on osseous development in young adults. Teeth development in both sexes was completely independent of BMI. Therefore, dental methods should be part of every age assessment.

## Introduction

Forensic age assessment in the living is an essential instrument for ensuring the rule of law [1–3]. Whenever the age of a person is unknown or there is reasonable doubt about the age statement of an individual, the most likely age or the exceeding of forensically relevant age limits can be evaluated [2]. Forensic age assessment is particularly important in view of the recently increasing migration movements [4]. This is related to a high number of individuals with inconclusive age information. Forensic age assessment can then be requested in order to guarantee age-appropriate care and constitutional procedures [2, 5].

In 2008, the international and multidisciplinary Study Group on Forensic Age Diagnostics published revised recommendations for age assessment in living adolescents and young adults [3]. In order to comply with these recommendations, an age assessment of a living individual should consist of a physical examination, a hand radiograph, and, in case of a completed development of the hand skeleton, either a conventional radiography or a CT examination of the medial clavicular epiphyses. In addition, an orthopantomogram and an examination of the dental status should be conducted [3]. Other skeletal regions have also been proposed for age assessment. In particular, the distal radius, the distal tibia, and the proximal femur have been described as potential regions of interest [6–10].

At present, great efforts are being made to replace the radiological examinations with radiation-free imaging procedures using MRI [6, 11–14]. It is therefore expected that the use of MRI of various body regions will be reflected in updated recommendations in the future.

In age assessment practice, the question arises which factors can influence the development of the characteristics examined. Socioeconomic status seems to influence the skeletal age, whereas tooth development is reported as unaffected by external factors [15–19].

The aim of this study is to assess to what extent the body mass index (BMI), as an indicator of the nutritional status of an individual, has an effect on age assessment. For this purpose, the association between body mass index and developmental stages of several features was investigated in a carefully sampled reference population.

## Materials and methods

This prospective cross-sectional study included 670 volunteers (334 females and 336 males) aged 12 to 24 years with known date of birth based on official documents. The 12-year age group, for example, included individuals aged between 12.00 and 12.99 years.

Ethics approval was granted by the Ethics Committee of the Medical Faculty of the University of Münster. After being duly informed, all study participants and/or their legal next of kin gave their written informed consent to take part in the study.

MRI scans were performed at the Translational Imaging Center (TRIC) operated by the Institute of Clinical Radiology at University Hospital Münster. Imaging was carried out using a 3.0 T magnetic resonance tomograph (Philips 3.0 T Achieva, gradient 80 mT/m; Philips Medical Systems, the Netherlands). A View Forum workstation (Philips Medical Systems, Netherlands) with a diagnostic monitor in a darkened room was used to evaluate the MRI images.

Based on the MRI scan, we evaluated the epiphysis of the distal radius, epiphysis of the medial clavicle, epiphysis of the distal femur, and epiphysis of the proximal tibia. The extent of ossification of the epiphyses was assessed using the established stage classifications by Schmeling et al. (2004) and Kellinghaus et al. (2010) [20, 21]. In addition, the status of development of the left lower third molars was examined. For this, the MRI scans of the corresponding teeth were evaluated according to the mineralization stages described by Dermirjian et al. (1973) [22, 23].

The MRI protocols and settings used are well proven in our institution and have already been published in previous publications [6, 8, 14, 24, 25].

In addition, all subjects were weighed on a calibrated scale and their height was measured. The body mass index (BMI) was then calculated from this data as the ratio of body weight in kilograms and the squared height in meters (kg/m^2^) [26].

The association between the assigned stage of the examined feature and BMI of the individual was assessed using linear regression analysis with BMI as a continuous variable. The association between BMI and an overall level of bone ossification, including all bone features together, was also examined. For the overall level of bone ossification, we added up the ossification levels of femur, tibia, radius, and clavicle. The minimum was 13 points and the maximum 32 points.

Since both BMI and the stages of development of the examined features physiologically increase from the age of 6 to adulthood, we adjusted all analysis for age to differentiate the true effect of BMI from the confounding effect of age [27, 28]. Only if BMI remained associated with stages of development in the age-adjusted analysis, BMI could be seen as an independent predictor of stages of development. For both BMI and the potential confounder age, we applied a fractional polynomial approach to allow for nonlinearity in the effect [29–31].

For the results of the linear regression analyses, the regression coefficients (*ß*), the 95% confidence intervals (CI), the coefficient of determination (*r*^2^), and *p* values are provided. While the regression coefficient *ß* with its confidence interval represents the strength of an association, *r*^2^ shows the proportion of variance in the data explained by the respective variables in the model. Data management and statistical analyses were performed in Stata, version 13.0 (Stata Corp LP).

## Results

**Fig. 1:**
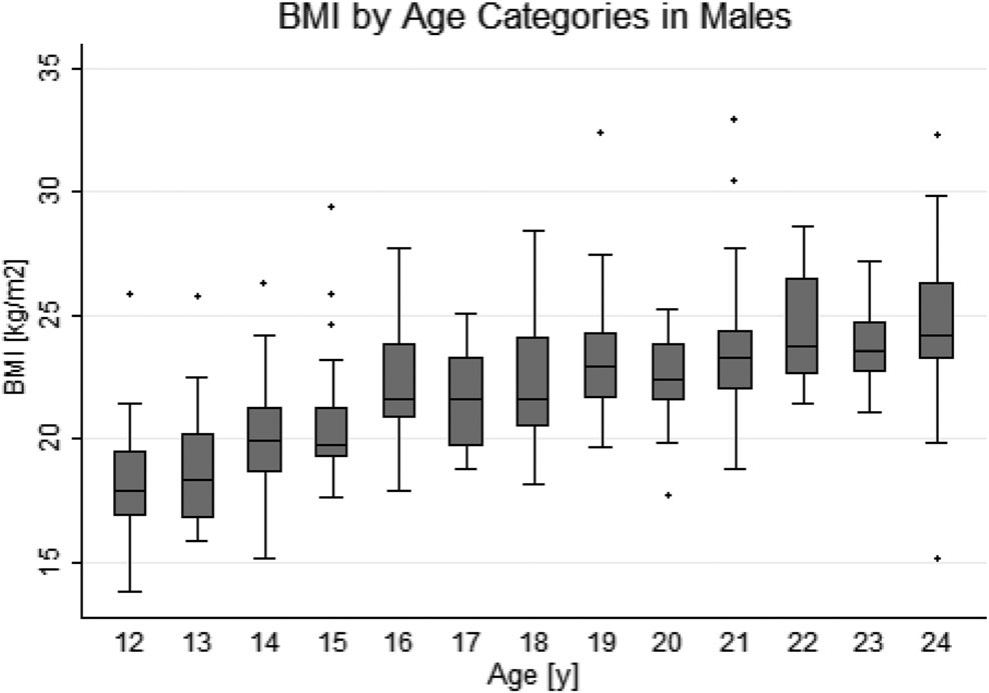
BMI by age categories in males

**Fig. 2:**
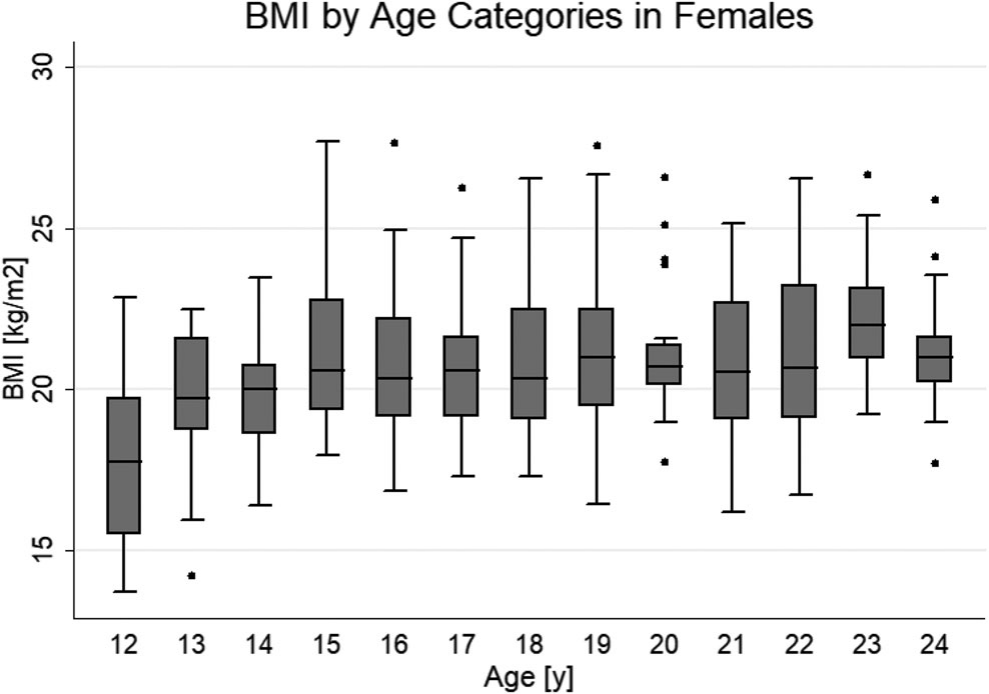
BMI by age categories in females

Due to various causes (e.g., discomfort in the scanner, continuous movements), not all examinations could be carried out successfully in all participants so that a total of 581 persons, 294 females and 287 males, could be included in the analysis (Table 1). Tables 2 and 3 show cohort baseline characteristics by sex and BMI. A total of 90 persons had a BMI < 18.5 kg/m^2^. The lowest BMI of 13.7 kg/m^2^ was found in a 12.88-year-old female. BMI values above 30 did occur only with a single case in females and only sporadically in males. The highest BMI of 35.1 kg/m^2^ was found in an 18.81-year-old female. Apart from this one female, all individuals with a BMI > 30 (*n* = 4) were males. The youngest male with a BMI > 30 kg/m^2^ was 19.2 years old. Sixty-six individuals had a BMI > 25 kg/m^2^; 44 of them were males and 22 females. Figures 1 and 2 show box plots of BMI across age groups from 12 to 24 years in males and females, respectively.

**Table 1:** Cohort composition by age and sex

Age, years	Female, *n*	Male, *n*	Total, *n*
12	16	21	37
13	13	27	40
14	24	18	42
15	27	23	50
16	29	25	54
17	23	25	48
18	27	26	53
19	29	24	53
20	22	22	44
21	24	25	49
22	22	24	46
23	19	9	28
24	19	18	37
Total, *n*	294	287	581

**Table 2:** Cohort baseline characteristics by sex. *SD*, standard deviation; *BMI*, body mass index; *IQR*, interquartile range

Characteristics	Total	Male	Female	*p* value
	*n* = 581	*n* = 287	*n* = 294	
Mean age (SD), years	18.41 (3.50)	18.23 (3.56)	18.59 (3.43)	0.21
Mean height (SD), cm	173.54 (10.28)	178.91 (10.62)	168.29 (6.57)	< 0.001
Mean weight (SD), kg	64.84 (13.44)	70.84 (14.50)	58.96 (9.06)	< 0.001
Mean BMI (SD), kg/m^2^	21.33 (2.90)	21.93 (3.15)	20.75 (2.49)	< 0.001
Median femur ossification (IQR)	8 (5, 8)	8 (4, 8)	8 (7, 8)	< 0.001
Median tibia ossification (IQR)	8 (6, 8)	8 (4, 8)	8 (7, 8)	< 0.001
Median radius ossification (IQR)	7 (5, 8)	7 (5, 8)	7 (7, 8)	0.005
Median clavicle ossification (IQR)	5 (2, 7)	5 (1, 7)	5 (2, 7)	0.018
Third molar development (IQR)	6 (5, 7)	6 (5, 7)	6 (5, 7)	0.068

**Table 3:** Cohort baseline characteristics by BMI. *SD*, standard deviation; *BMI*, body mass index; *IQR*, interquartile range

Characteristics	Total	BMI 1st tertile	BMI 2nd tertile	BMI 3rd tertile	*p* value
	*n* = 581	*n* = 196	*n* = 197	*n* = 187	
Females (%), *n*	294 (50.60)	116 (59.20)	112 (56.90)	65 (34.80)	< 0.001
Mean age (SD), years	18.41 (3.50)	16.57 (3.38)	18.66 (3.18)	20.06 (3.02)	< 0.001
Mean height (SD), cm	173.54 (10.28)	169.31 (10.42)	173.99 (9.82)	177.51 (8.87)	< 0.001
Mean weight (SD), kg	64.84 (13.44)	53.05 (8.56)	64.26 (7.80)	77.81 (10.26)	< 0.001
Mean BMI (SD), kg/m^2^	21.33 (2.90)	18.39 (1.34)	21.15 (0.68)	24.62 (1.92)	< 0.001
Median femur ossification (IQR)	8 (5, 8)	5 (4, 8)	8 (5.5, 8)	8 (8, 8)	< 0.001
Median tibia ossification (IQR)	8 (6, 8)	7 (4, 8)	8 (6, 8)	8 (8, 8)	< 0.001
Median radius ossification (IQR)	7 (5, 8)	5 (4, 7)	7 (5.5, 8)	8 (7, 8)	< 0.001
Median clavicle ossification (IQR)	5 (2, 7)	2 (1, 6)	5 (2, 7)	6 (4, 7)	< 0.001
Third molar development (IQR)	6 (5, 7)	5 (4, 6)	6 (5, 7)	7 (6, 8)	< 0.001

CDC (US Centers for Disease Control and Prevention) considers that being in the percentile 5% of BMI distribution indicates low weight and 95 percentile indicates overweight [32, 33]. Related to the age-specific reference values of the CDC, the following pathological BMI values were found in the collective: four 12-year-olds (3 females, 1 male) had a BMI < 5th percentile (BMI 14.5) and thus in the pathological range. One 17-year-old and one 18-year-old female each had a BMI < 17.5 and thus below age-appropriate 5th percentile. BMI values > 23.5 (reported by CDC as 95th percentile of 12-year-olds) were not detected in the collective until the age of 16.21 years. Four persons under 18 years (2 females, 2 males) showed a BMI > 27.5 (reported by CDC as 95th percentile for 17-year-olds) and thus a pathologically increased BMI. For all these 4, the values were below BMI = 30 and thus in the physiological range if only the reference values for adults were taken into account. A total of 18 persons (5 females, 13 males) showed a BMI > 27.5. Ten of these 18 persons were over 20 years old (95th percentile for 20-year-olds according to CDC: BMI > 31).

For the investigation of the influence of BMI on the characteristics, a distinction was also made between the crude and age-adjusted approaches for the assessment of the statistical findings (Table 4).

**Table 4:** Results for the effect of BMI on all features stratified by sex (linear regression adjusted for age using a fractional polynomial). *ß*, coefficient of correlation; *CI*, confidence interval; *r*^*2*^, coefficient of determination

	Crude/adjusted for age	Male				Female			
		*ß*	95% CI	*p* value	*r*^2^	*ß*	95% CI	*p* value	*r*^2^
Total bone ossification	Crude	1.39	1.12; 1.67	< .001	0.32	0.74	0.47; 1.02	< .001	0.10
	Adjusted	0.08	− 0.05; 0.22	0.23	0.89	0.11	0.02; 0.21	0.021	0.90
Femur ossification	Crude	0.32	0.27; 0.38	< .001	0.32	0.18	0.12; 0.25	< .001	0.10
	Adjusted	0.02	− 0.01; 0.06	0.18	0.83	0.03	− 0.00; 0.06	0.079	0.81
Tibia ossification	Crude	0.31	0.26; 0.36	< .001	0.33	0.17	0.11; 0.23	< .001	0.11
	Adjusted	0.02	− 0.01; 0.05	0.14	0.85	0.03	0.00; 0.06	.021	0.83
Radius ossification	Crude	0.30	0.25; 0.35	< .001	0.33	0.17	0.11; 0.22	< .001	0.11
	Adjusted	0.03	0.00; 0.06	0.042	0.83	0.05	0.02; 0.08	.001	0.78
Clavicle ossification	Crude	0.44	0.34; 0.54	< .001	0.27	0.24	0.13; 0.34	< .001	0.07
	Adjusted	0.00	− 0.06; 0.05	0.91	0.85	0.04	− 0.01; 0.09	0.13	0.82
Third molar development	Crude	0.16	0.13; 0.20	< .001	0.26	0.10	0.05; 0.14	< .001	0.07
	Adjusted	0.00	− 0.04; 0.04	0.99	0.77	0.03	− 0.02; 0.07	0.31	0.62

In the univariable analysis, BMI was strongly associated with higher stages on all feature scales (*p* < 0.01, Table 4). The proportion of variance in feature scale stages explained by BMI (*r*^2^) varied between 0.26 and 0.33 for males but was considerably lower for females (0.07–0.11). *ß* was between 0.17 and 0.44 depending on the characteristic.

However, after adjusting for age, beta coefficients decreased considerably. Significant results with the age-adjusted approach could only be achieved for the ossification of the distal radius (*p* = 0.042 in males and *p* = 0.001 in females) and for the ossification of the proximal tibia in females (*p* = 0.021). Even for these cases, however, the adjusted beta was rather small (< 0.06). *r*^2^ here was between 0.78 and 0.85.

When all bone characteristics were considered together, beta coefficients were again substantially lower in the age-adjusted (males: *ß* = 0.08; females: *ß* = 0.11) than in the crude analysis (males: *ß* = 1.39; females: *ß* = 0.74). *r*^2^ was 0.10 (females) and 0.32 (males) for the crude analysis and 0.89 (males) and 0.90 (females) for the age-adjusted approach.

For the development of the third molars, beta coefficients (*ß* = 0.16 in males and *ß* = 0.10 in females) were already lower in the univariable analysis than for all bone characteristics. Looking at the adjusted approach, the influence of BMI on tooth development is again reduced or eliminated (males: *ß* = 0.00; females: *ß* = 0.03). Regarding the development of the third molars, *r*^2^ was 0.07 (females) and 0.26 (males) for the crude analysis and 0.77 (males) and 0.62 (females) for the age-adjusted approach.

## Discussion

In our study BMI was associated with the stages of skeletal maturation before taking age into account. However, if BMI was adjusted for age, the effect (*ß*) was no longer apparent indicating that BMI in this context served only as a proxy for age.

The present study was intended to clarify the influence of the nutritional status on skeletal maturation and third molar mineralization. Against the background of the dramatically increasing incidence of overweight and obese children, the research question of this study is of great practical relevance [34].

Thus, the present study aimed to investigate the influence of BMI in a normal population with a normally distributed BMI, without pre-selected BMI values. Although the cohort was composed originally to examine a different research question, no distortion in the presentation of the BMI can be assumed in principle. In this way, the influence of BMI on the characteristics could be investigated as it is reflected in the everyday procedures of forensic age assessment.

We innovatively used MRI technology to examine the individual stages of development for the present study. We did not focus on verifying the validity of the imaging procedure because the use of MRI has already been evaluated in various studies [6, 7, 14, 25, 35–38].

The age-dependent behavior of BMI is long known and was confirmed in a large study conducted in Germany in 2003–2006 with 17,641 individuals aged 0–17 years [39]. The BMI of infants and young children increased continuously during the first months of life and reached a maximum at about an age of 9 months, which was slightly higher for males than for females. Then, the BMI decreased steadily up to an age of about 5 years. In an international comparison of the WHO reference values, children in a 2010 German study showed a higher BMI in all age groups except for the first 9 months [39, 40]. The physiological BMI also continues to increase in adults. However, these changes are no more as noticeable as in the development phase. The physiological BMI only falls slightly again at a high age of about 70 years and older [27]. This age dependency of BMI must be known to the examiners in order to detect cases of pathological BMI.

Several studies have shown that during prepubertal years, obese children have higher height velocity and accelerated bone age compared with lean subjects [41, 42].

In 2001, Russel et al. wanted to examine the connection between the differences in the skeletal age of two ethnic groups. They examined 252 African American and Caucasian children aged 5–12 years in the USA. Russel et al. summarized that skeletal age was more advanced in African American than Caucasian children and was significantly related to body mass [43].

Artioli et al. studied in 2019 the influence of BMI on skeletal age [44]. They examined a total of 777 children aged between 5 and 17 years in Brazil. The individuals were divided into 3 groups (eutrophic, overweight, and obese), and the skeletal age of the groups was compared. The Greulich-Pyle and BoneXpert methods were used to determine the skeletal age. The authors found that obese boys presented advanced bone age compared with eutrophic or overweight boys, with both the Greulich-Pyle and BoneXpert methods. There was no significant difference in bone age between eutrophic and overweight boys regardless of the method used to determine bone age. In girls, there was bone age advancement in both obese and overweight girls when compared with eutrophic girls. However, this observation was present only with the Greulich-Pyle method. When the BoneXpert method was used, the bone age advancement was identified in obese girls when compared with eutrophic or overweight girls. The authors identified a clear trend towards the association between BMI and skeletal development [44]. The fact that this clarity could not be confirmed in the results of the present study may be due to the preselection of the individuals in the cited study. In our study, BMI was to a larger extent in a normal range.

Soares et al. investigated in 2019 the effect of BMI on hand skeleton age and found a strong association between BMI and advanced skeletal age, but only in females and without taking age into account [41].

The cause of accelerated skeletal maturation in overweight children has not been conclusively clarified. In 2017, de Groot et al. stated an increased level of DHEAS (dehydroepiandrosterone-sulfate) in overweight or obese children as an explanation for the advanced skeletal age [45].

Other factors which have been suggested to be involved in the accelerated growth in obese children include increased leptin and insulin levels, insulin-like growth factor (IGF)-1, IGF-binding protein (IGFBP)- 1 and GH-binding proteins (GHBP) [42].

Other authors found that bone age is more advanced in obese children with hyperinsulinemia and insulin resistance. They therefore assume that insulin is involved in bone development [46, 47]. This means that an effect of BMI on bone age might only exist in case of pathological (diabetic) obesity.

In addition to these approaches, which postulate a changed hormone situation as the cause of a potential effect of BMI on bone development, another approach could also explain the association: the well-established effect of mechanical loading conferred by body weight on bone formation. This approach focuses on the micro-metabolism of the bone due to the increased body weight leading to higher mechanical load [48]. This effect cannot be deduced from the results of the present study: tibia and femur as weight-bearing bones have no stronger association to BMI than other bones.

It is important to note that in all the studies cited, mainly children (< 18 years) were examined. Thus, the age in the cohorts is not in line with the one of the present study. In persons older than 18 years, the effect of BMI on bone age was indeed rather small if at all present.

The assessment of third molar mineralization in the orthopantomogram is part of an age assessment corresponding to the recommendations of the AGFAD [3]. This is not least the case since the relevant literature assumes that tooth development is largely independent of external factors [19].

The influence of the BMI on the development of the third molar was extremely low with betas of 0.00 (male) and 0.03 (female). Even in the crude analysis, beta values were considerably lower than for bone development. This is further evidence that tooth development is independent of external factors and seems to be mainly genetically determined. The consideration of tooth development can therefore still be regarded as a very important element of age assessment procedures.

In conclusion, our study found little to no effect of BMI on bone development after taking age into account. Teeth development in both sexes was completely independent of BMI—therefore, dental methods should be part of every age assessment. In the literature, advanced skeletal age has been described especially for overweight and obese young children, for reasons unknown today. Most likely, however, pathological changes in hormone balance play a decisive role. Caution is therefore required in age estimation procedures in these cases.
